# Systematic and functional analysis of non-specific lipid transfer protein family genes in sugarcane under *Xanthomonas albilineans* infection and salicylic acid treatment

**DOI:** 10.3389/fpls.2022.1014266

**Published:** 2022-10-05

**Authors:** Juan Li, Jian-Ying Zhao, Yang Shi, Hua-Ying Fu, Mei-Ting Huang, Jian-Yu Meng, San-Ji Gao

**Affiliations:** National Engineering Research Center for Sugarcane, Fujian Agriculture and Forestry University, Fuzhou, China

**Keywords:** sugarcane, nsLTP, expression analysis, defense response, *Xanthomonas albilineans*, salicylic acid

## Abstract

Plant non-specific lipid transfer proteins (nsLTPs) are small basic proteins that play a significant regulatory role in a wide range of physiological processes. To date, no genome-wide survey and expression analysis of this gene family in sugarcane has been performed. In this study we identified the nsLTP gene family in *Saccharum spontaneum* and carried out expression profiling of *nsLTPs* in two sugarcane cultivars (*Saccharum* spp.) that have different resistance to leaf scald caused by *Xanthomonas albilineans* (*Xa*) infection. The effect of stress related to exogenous salicylic acid (SA) treatment was also examined. At a genome-wide level, *S. spontaneum* AP85-441 had 71 *SsnsLTP* genes including 66 alleles. Tandem (9 gene pairs) and segmental (36 gene pairs) duplication events contributed to *SsnsLTP* gene family expansion. Five SsnsLTP proteins were predicted to interact with five other proteins. Expression of *ShnsLTPI.8/10/Gb.1* genes was significantly upregulated in LCP85-384 (resistant cultivar), but downregulated in ROC20 (susceptible cultivar), suggesting that these genes play a positive regulatory role in response of sugarcane to *Xa* infection. Conversely, *ShnsLTPGa.4/Ge.3* appears to act as a negative regulator in response *Xa* infection. The majority (16/17) of tested genes were positively induced in LCP85-384 72 h after SA treatment. In both cultivars, but particularly in LCP85-384, *ShnsLTPIV.3/VIII.1* genes were upregulated at all time-points, suggesting that the two genes might act as positive regulators under SA stress. Meanwhile, both cultivars showed downregulated *ShnsLTPGb.1* gene expression, indicating its potential negative role in SA treatment responses. Notably, the *ShnsLTPGb.1* gene had contrasting effects, with positive regulation of gene expression in response to *Xa* infection and negative regulation induced by SA stress. Together, our results provide valuable information for elucidating the function of *ShnsLTP* family members under two stressors and identified novel gene sources for development of sugarcane that are tolerant of environmental stimuli.

## Introduction

Plant non-specific lipid transfer proteins (nsLTPs) have lipid binding activity that mediates transfer of various phospholipids, acyl groups, and fatty acids between biological membranes (Liu et al., 2015; Salminen et al., 2016; Missaoui et al., 2022). Plant nsLTPs are characterized by small molecular weight (6.5–10.5 kDa) and a backbone with an eight-cysteine motif (ECM: C-Xn-C-Xn-CC-Xn-CXC-Xn-C-Xn-C) (Wang et al., 2012; Salminen et al., 2016). Furthermore, nsLTPs have four α-helices and are stabilized by four conserved disulfide bridges (Salminen et al., 2016; Missaoui et al., 2022). The cysteine residues are engaged in these four disulfide bonds to stabilize the tertiary structure of the hydrophobic cavity and afford size plasticity that allows for binding of different lipids and hydrophobic compounds as documented in *in vitro* assays (Boutrot et al., 2008; Liu et al., 2015).

The classification and naming of LTPs has developed over the past two decades. Kader (1996) initially reported that plant nsLTPs could be classified into two major types, nsLTP1 (9 kDa) and nsLTP2 (7 kDa), based on the molecular size of the mature protein. Subsequently, Boutrot et al. (2008) categorized nsLTPs from three plant species into nine types (I–IX) based on sequence similarity and/or phylogenetic clustering. Edstam et al. (2011) modified this nsLTP classification system according to intron positions, presence of glycosylphosphatidylinositol (GPI) modification sites, Cys spacing in the ECM, and sequence similarity, which resulted in five major types (LTP1/2 and LTPc/d/g) and five minor types (LTPe/f/h/j/k). Salminen et al. (2016) developed a well-defined and informative naming system for LTPs and proposed classification of LTPs as conventional type (i.e., LTP1/2) and nine other types (i.e., LTPc/d/e/f/g/h/j/k/x). Recently, Fleury et al. (2019) developed an updated classification system of the nsLTP superfamily (Types I–VI, VIII, IX, and XI) in angiosperm, gymnosperm, lycophyte, and bryophyte species based on the length, composition, and structure of 797 nsLTP proteins.

Plant nsLTPs are pathogenesis-related proteins and mediate diverse biological functions such as seed development and germination, cutin and wax metabolism, and defense signal transduction, as well as stress response and regulation (Missaoui et al., 2022). Under biotic stress, plant nsLTPs play dual roles in defense responses. In wheat, *TaDIR1-2* (nsLTP-Type II group) is negatively regulated in resistance to the fungal pathogen *Puccinia striiformis* f. sp. *tritici* that causes wheat stripe rust (Ahmed et al., 2017). Overexpression of *AtLTP4.4* in transgenic wheat significantly increased resistance to blight caused by the fungus *Fusarium graminearum* (McLaughlin et al., 2021). The *StLTP10* gene acts as a positive regulator for resistance of potato (*Solanum tuberosum*) to *Phytophthora infestans* (Wang et al., 2021). *NbLTP1* from *Nicotiana benthamiana* promotes bamboo mosaic virus accumulation (Chiu et al., 2020) and *StLTP6* from potato facilitated viral infection (Shang et al., 2022). *GhnsLTPsA10* from cotton (*Gossypium hirsutum*) positively enhanced resistance to fungi, but negatively mediates insect resistance in transgenic *Arabidopsis* and cotton with gene silencing (Chen et al., 2021). On the other hand, plant nsLTPs act as essential regulators against abiotic stressors. For example, *TaDIR1-2* expression was significantly upregulated in wheat seedlings treated with salicylic acid (SA) or low temperature (Ahmed et al., 2017). Transgenic potato lines overexpressing *StnsLTP1* displayed enhanced tolerance of heat, water-deficit, and salt stresses (Gangadhar et al., 2016). *OsLTPL159* is associated with cold tolerance at the early seedling stage in *japonica* rice (Zhao et al., 2020). Transcriptome analysis showed that *BnLTP* genes were involved in *Brassica napus* responses to multiple abiotic stresses such as heat, drought, NaCl, cold, indoleacetic acid, and abscisic acid (ABA) (Xue et al., 2022).

In sugarcane, expression of a putative homolog of LTPs was differentially induced in a sugarcane genotype that is resistant to eyespot disease caused by *Bipolaris sacchari* infection (Borrás-Hidalgo et al., 2005). Expression levels of three *nsLTP* transcripts were increased in sugarcane plants under water stress induced by irrigation suppression (Rodrigues et al., 2011). A *nsLTP* gene isolated from a sugarcane cDNA library was upregulated in IBP8518 (resistant to brown rust), but not in B4362 (susceptible to brown rust) cultivars at 5–7 days after inoculation with *Puccinia melanocephala* (Oloriz et al., 2012). Meanwhile, *ScNsLTP* gene played an opposing role in sugarcane seedlings under SA and methyl jasmonate (MeJA) treatments, but was positively upregulated under chilling and osmotic stress (Chen et al., 2017). Recently, a nsLTP (Cluster-4871.183445) from sugarcane was shown to act as a potentially positive regulator of the defense response to *Xanthomonas albilineans* (*Xa*), the bacterial pathogen of leaf scald (Meng et al., 2020). Furthermore, numerous nsLTP genes were identified in sugarcane in response to infection with beneficial bacteria (*Gluconacetobacter diazotrophicus*) and *Acidovorax avenae*, the causative bacterial pathogen for red stripe disease, as well as water depletion (de Oliveira Silva et al., 2022). Apart from these observations, systematic identification and expression analysis of the nsLTP gene family in sugarcane under diverse environmental stress is poorly understood.

Leaf scald caused by the bacterial pathogen (*Xa*) is one of three main bacterial diseases of sugarcane, and causes substantial loss of yield (Lin et al., 2018). SA is recognized as a key plant hormone required for host resistance responses to many pathogens (Ding and Ding, 2020), although systematic and functional analyses of this gene family in sugarcane under *Xa* infection and SA treatment have not been carried out. The three objectives of the present study are to conduct: (i) genome-wide identification and characterization of nsLTP gene family members in *Saccharum spontaneum* AP85-441; (ii) systematic analysis of expression profiling of nsLTPs in two sugarcane cultivars having contrasting resistance to leaf scald under *Xa* infection and SA treatment; and (iii) molecular analyses to generate important gene resources for genetic improvement of stress tolerance in sugarcane.

## Materials and methods

### Genomic sequence retrieval and identification of non-specific lipid transfer protein gene family members

To identify candidate nsLTPs, the genomic sequence of the AP85-441 of *S. spontaneum* clone was retrieved from the online sequence made available by Dr. Ming’s Laboratory^1^ (Zhang et al., 2018) and the Hidden Markov Model (HMM) profile of plant lipid transfer proteins (PF00234) was retrieved from the Pfam database.^2^ The HMM profile PF00234 was used as a query to search against the AP85-441 protein database using default parameters of HMMER software (Finn et al., 2011) and a cut-off value of 1e^–10^. Published *Arabidopsis* nsLTP sequences were also retrieved and used as a query to identify *S. spontaneum* nsLTPs with BLASTp^3^ searches having a cutoff value of 1e^–3^. Then, the candidate nsLTP sequences were manually checked and proteins lacking an ECM domain were removed. To exclude possible grain storage proteins, a BLASTp search was carried out on the protein sequence of 2S-albumin (At2S1 to At2S4) (Guerche et al., 1990) and alpha-amylase inhibitor (RATI) (Strobl et al., 1998) within the candidate *S. spontaneum* nsLTP proteins (termed as SsnsLTPs). Lastly, SsnsLTPs identified in AP85-441 were named according to the convention proposed by Boutrot et al. (2008).

### Phylogenetic analysis, chromosomal location, and gene duplication analysis

Multiple sequence alignment of nsLTP proteins from *S. spontaneum* was performed using the ClustalW program in MEGA 7.0 software (Kumar et al., 2016). The phylogenetic tree of aligned sequences was constructed using MEGA7.0 software with the neighbor-joining method and bootstraps of 1,000 replicates. MapChart^4^ was used to map the *SsnsLTPs* on AP85-441 chromosomes. TBtools (Toolbox for biologists) v0.6655 (Chen et al., 2020) was used to identify gene duplication events.

### *In silico* sequence analysis

SsnsLTP protein isoelectric points were calculated using the ExPASy Proteomics Server.^5^ Sequence Manipulation Set^6^ was used to determine the molecular weights of the proteins. The ProtParam online analysis tool^7^ was used to determine the theoretical pI (isoelectric point), instability index (II), aliphatic index (AI), and grand average of hydropathicity (GRAVY). The exon-intron distribution pattern of *SsnsLTPs* was searched on GSDS 2.0.^8^ MEME5.4.1^9^ was used to determine conserved motifs, followed by visualization with TBtools v0.6655. Prediction of protein–protein interactions among SsnsLTPs and with other sugarcane proteins was performed according to their orthologs in *Sorghum bicolor* using the STRING database.^10^

### Plant materials

The two sugarcane cultivars, LCP85-384 and ROC20, which are resistant and susceptible to leaf scald, respectively, were grown in a sugarcane nursery in Fuzhou, China. Single buds cut from healthy plant stalks were immersed under flowing tap water overnight at room temperature and then were treated with hot water at 50°C for 2 h. The disinfected buds were cultured for germination and grown in commercial nutrient soil (Pindstrup Mosebrug A/S, Fabriksvej, Denmark) in an artificial incubator for 28 days at 28°C, relative humidity 60%, and a 16/8 h light/dark cycle.

### Bacterial inoculation and salicylic acid treatment

Sugarcane plants were inoculated with bacterial suspension cells (10^8^ CFU/ml) of *Xa* strain Xa-FJ1 (Zhang et al., 2020) using the cutting leaf method and samples were collected from the inoculated plants at 0, 24, 48, and 72 h post inoculation (hpi). Control plants were inoculated with sterile XAL medium (liquid XAS) (Davis et al., 1994). Additional plants were sprayed with exogenous SA (0.1 mmol/L SA containing 0.01% Tween-20) and samples were collected at 0, 6, 12, and 24 hour post treatment (hpt). Three biological replicates (six plants each) were used for the individual time points in both experiments. All treated plants were grown as described above.

### Quantitative RT-PCR assay

Total RNA from leaves was extracted using a TRIzol reagent kit (Invitrogen, USA). The cDNA was synthesized using HiScript II Q RT SuperMix with a qPCR (+gDNA wiper) reverse transcription kit (Vazyme Biotech Co., Ltd., Nanjing, China). The relative transcription level of nine selected *nsLTP* gene*s* in the sugarcane cultivars (termed *ShnsLTPs*) was determined using ChamQ Universal SYBR qPCR Master Mix (Vazyme Biotech). *ShnsLTP* gene sequences were collected from RNA-seq data, and gene-specific primers were designed using Primer Premier 6 software^11^ (Supplementary Table 1). The SYBR Green dye method was used for qPCR assays with 20 μl reaction volumes that included 0.4 μl of both forward and reverse primers (10 μM), 1 μl cDNA, 10 μl 2× ChamQ SYBR qPCR Master Mix, and 8.2 μl nuclease-free water. A qPCR standard program was conducted on a QuantStudio 3 fluorescence quantitative PCR instrument (Applied Biosystems, CA, USA) with initial denaturation at 95°C for 30 s, followed by 40 cycles of 95°C for 5 s and 62°C for 30 s. Three biological replicates and three technical replicates were used for all samples. The housekeeping gene glyceraldehyde-3-phosphate dehydrogenase (GAPDH) was used as a reference gene and *ShnsLTP* expression levels from different samples were normalized using the 2^–ΔΔCt^ method (Livak and Schmittgen, 2001).

### Statistical analysis

The relative expression levels of each gene were analyzed by one-way ANOVA and the SNK (Student–Newman–Keuls) test was used to determine significant differences (*p* ≤ 0.05) among means. All analyses were carried out using SAS version 8.1 (SAS Institute, Cary, NC, USA).

## Results

### Identification and properties of SsnsLTP proteins

Among 158 truncated candidate SsnsLTP sequences, 87 protein sequences were discarded including 63 protein sequences that had incomplete ECM domains or lacked signal peptides, as well as 12 proline-rich proteins, 6 α-amylase/trypsin inhibitors and 6 2S albumin storage proteins. A total of 71 *SsnsLTP* genes were obtained and classified into two categories according to sequence similarity and the presence or absence of a GPI-anchor. The 71 *SsnsLTPs* were also divided into 13 types (I–VIII and Ga-Ge) with varying numbers of genes in each type. Type I had the most genes (22) and Types VI and VIII each had only one gene (Supplementary Table 2).

**FIGURE 1:**
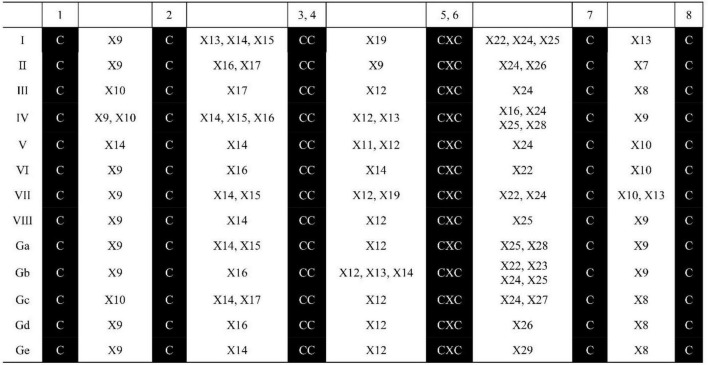
Characteristics of ECM structure for nsLTP family genes identified in *S. spontaneum* AP85-441. “X” represents any amino acid, and the Arabic number beside “X” represents the number of amino acid residues.

The ECM structural features in the 71 identified SsnsLTP proteins are summarized in Figure 1. These SsnsLTP proteins possessed unique ECM structural features, while those within GPI-anchor sites had the conserved C^4^X_12_C^5^ motif except for proteins in class Gb, which had three kinds of motifs (C^4^X_12_C^5^, C^4^X_12–14_C^5^, and/or C^4^X_14_C^5^). SsnsLTP-I proteins had two conserved motifs: C^4^X_19_C^5^ and C^7^X_13_C^8^. SsnsLTP-Gb, –Ga, –IV, and –VIII class proteins contained the motif C^7^X_19_C^8^, and those in Class SsnsLTP-III, –VII, –GC, and –Gd classes possessed the C^4^X_12_C^5^ motif. SsnsLTPV contained the C^1^X_14_C^2^ motif and were clustered in type V (SsnsLTPV).

All identified *SsnsLTP* genes encoded proteins having varying numbers of amino acids, which ranged from 105 to 714. The protein molecular weights were between 10,895.75 and 76,418.3 Da. The isoelectric points (4.41–10.08), instability indexes (29.02–83.71), aliphatic indexes (73.24–107.19), and GRAVY (−0.02 to 0.64) also varied among the SsnsLTP proteins (Supplementary Table 2).

### Phylogenetic relationships of SsnsLTPs

**FIGURE 2:**
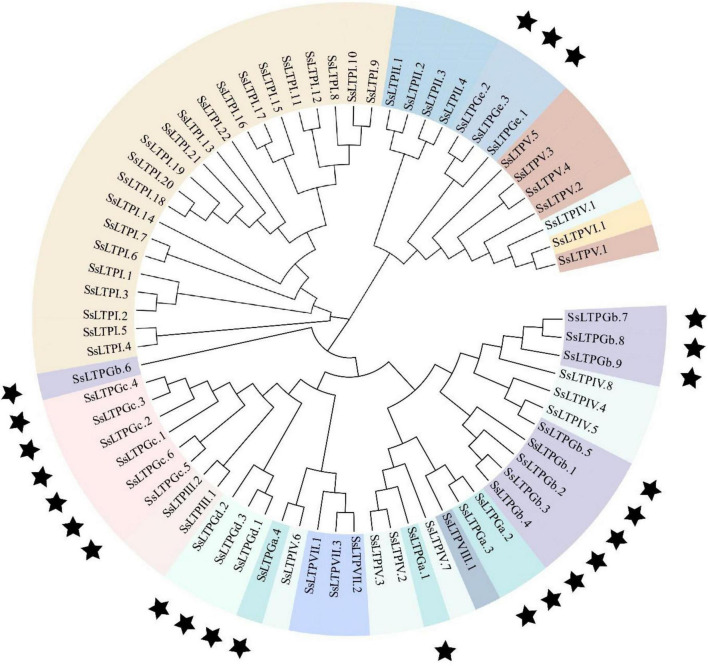
Phylogenetic tree of 71 *SsnsLTP* genes identified in *S. spontaneum*. The tree was constructed using the neighbor-joining (NJ) method with 1,000 bootstrap replicates. Genes shaded with the same color are the same type and black stars indicate G groups having a GPI gene.

To better understand the evolutionary relationship among SsnsLTPs, a phylogenetic tree was constructed using the 71 SsnsLTP protein sequences (Figure 2). The phylogenetic grouping of the SsnsLTP proteins was essentially identical to the classification based on ECM structural features. All 22 *SsnsLTPs* from class I (SsnsLTPI) clustered into a unique phylogenetic clade. Meanwhile, some *SsnsLTPs* from different classes clustered into the same phylogenetic clade. For example, 16 *SsnsLTPs* from four classes (SsnsLTPs-III, –VII, –GC, and –Gd), 19 *SsnsLTPs* from four classes (SsnsLTPs-IV, –VIII, –GB, and –Ga), and 14 *SsnsLTPs* from four classes (SsnsLTPs-II, –Ge, –V, and –VI) were grouped in three phylogenetic clades.

### Gene structure analysis of SsnsLTPs

**FIGURE 3:**
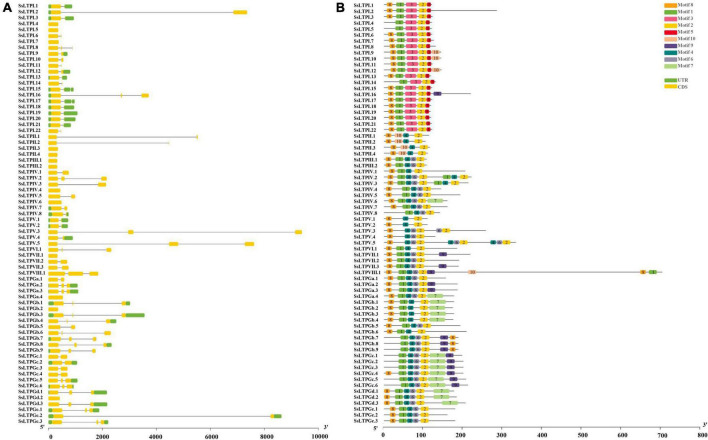
Gene structure **(A)** and conserved motifs **(B)** among 71 *SsnsLTPs* genes in *S. spontaneum*. **(A)** Exons and untranslated regions are indicated by yellow and green boxes, respectively, and thin gray lines represent introns. The scale bar at the bottom represents the number of nucleotides. **(B)** Conserved motifs (1–10) identified using the MEME online database are represented by different color boxes. The scale bar at the bottom represents the number of base pairs (bp).

Analysis of intron-exon structure and conserved motifs of all 71 *SsnsLTP*s showed that *SsnsLTPGa.4/Gb.2/Gd.2*, *SsnsLTPI.4/5/7*, *SsnsLTPII.3/4*, *SsnsLTPIII.1/2*, *SsnsLTPIv.4/6*, *SsnsLTPv.1* had only one exon and lacked both introns and a UTR structure. Other *SsnsLTP*s had 1–2 introns, 1–3 exons, and UTR structures (Figure 3). *SsnsLTPV.3* had the longest intron structure, followed by *SsnsLTPV.5* and *SsnsLTPGe.2*. All SsnsLTP proteins had Motif 2, while Motif 1 was found in most *SsnsLTPs* except for four genes in SsnsLTPII and five genes in SsnsLTPV. Motif 4 was observed in all SsnsLTP types except for *SsnsLTPI*. Motif 8 was present in 13% (9/71) of *SsnsLTPs*. Notably, Motif 3 and 5 were present only in SsnsLTPI, while Motif 10 was present only in SsnsLTPII. The same motif often occurred two or three times in a gene, as seen for *SsnsLTPV2/3* (double Motif 1, 2, and 4), *SsLTPV.3* (double Motif 2 and 4), *SsLTPVIII.1* (double Motif 1), and *SsLTPV.5* (triple Motif 2, 4, and 6).

### Chromosome location and gene duplication analysis of SsnsLTPs

**FIGURE 4:**
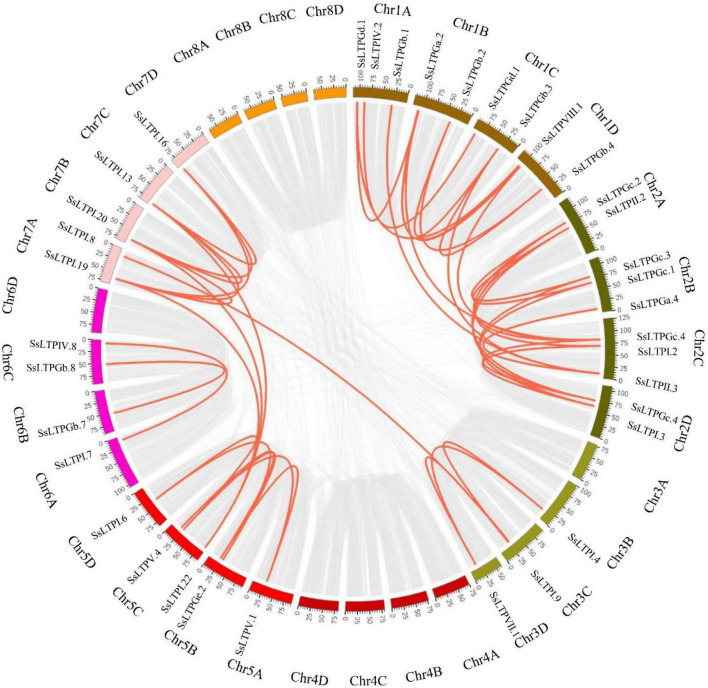
Gene duplication relationships of *SsnsLTP*s in the *S. spontaneum* AP85-441 genome. Gray and red lines represent all genes and *SsnsLTP*, respectively, that were duplicated in the genome. Chromosome names are displayed in the outermost ring.

Among all 71 *SsnsLTPs*, 92.9% (66/71) were derived from 23 *SsnsLTPs* with 1–4 alleles, while five *SsnsLTPs* lacked alleles. These *SsnsLTPs* localized on 23 chromosomes of *S. spontaneum* AP85-441 and had an uneven distribution among the different chromosomes. Only one *SsnsLTP* gene was present on Chromosomes 5D, 6A, 6D, and 7D. Two *SsnsLTP* genes occurred on chromosomes 2A, 3D, 5A, 5B, 6B, and 6C. Three or more *SsnsLTP* genes appeared on the other 13 chromosomes (Supplementary Figure 1). Notably, 63% (45/71) of *SsnsLTPs* underwent gene duplication events, including nine tandem-duplicated gene pairs and 36 segmental-duplicated gene pairs (Figure 4).

### Protein–protein interaction network among SsnsLTPs

**FIGURE 5:**
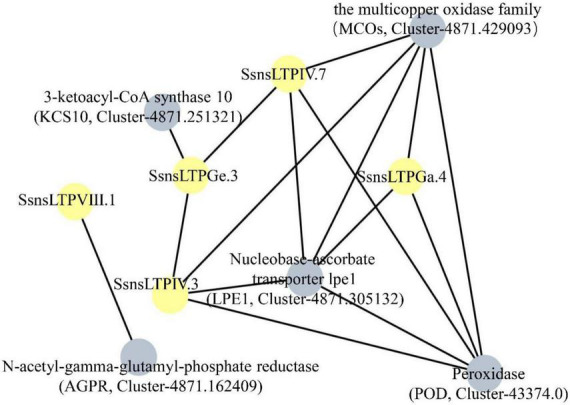
Prediction of interaction network among five SsnsLTPs according to their *S. bicolor* orthologs. Five SsnsLTPs and five interacting proteins are represented by gray and yellow nodes, respectively.

Five SsnsLTP proteins (SsnsLTPIV.3/IV.7/VIII.1/Ga.4/Ge.3) were predicted to interact with five different proteins including the multicopper oxidase family (MCOs) (Cluster-4871.429093), 3-ketoacyl-CoA synthase-10 (KCS10) (Cluster-4871.251321), nucleobase-ascorbate transporter lpe1 (LPE1) (Cluster-4871.305132), N-acetyl-gamma-glutamyl-phosphate reductase (AGPR) (Cluster-4871.162409), and peroxidase (POD) (Cluster-43374.0) (Figure 5). Additionally, SsnsLTPGe.3 interacted with SsnsLTPIV.3 and SsnsLTPIV.7. Among the ten interacting proteins, LPE1, POD, and MCOs likely played key roles in the protein-protein interaction network, as evidenced by their interactions with five SsnsLTP proteins each, including three SsnsLTPs (SsnsLTPIV.3/IV.7/Ga.4). Notably, MCOs and POD interacted with each other.

### ShnsLTP expression patterns in response to *Xanthomonas albilineans* infection

**FIGURE 6:**
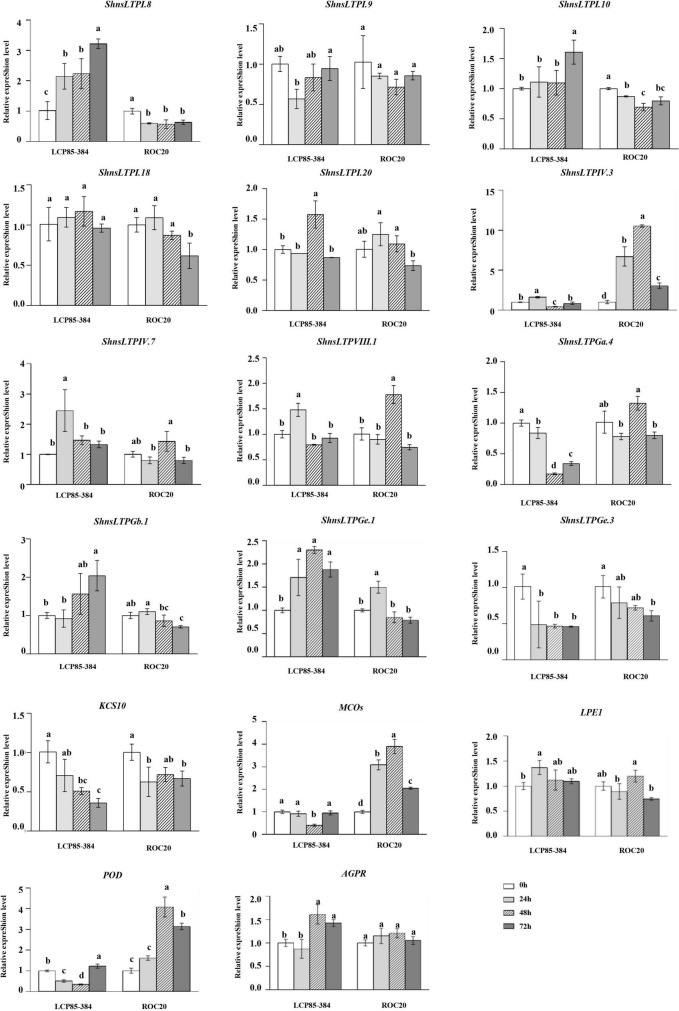
Transcript expression levels of 12 *ShnsLTPs* and 5 interacting protein genes in LCP85-384 (resistant to leaf scald) and ROC20 (susceptible to leaf scald) sugarcane cultivars after *X. albilineans* infection. The bars indicate mean ± SD (*n* = 9). The same letters indicate no significant difference (*p* > 0.05).

Transcript expression profiling by RT-qPCR assay of 12 *ShnsLTPs* and five genes for the abovementioned interacting proteins were investigated in two sugarcane cultivars LCP85-384 (resistant to leaf scald) and ROC20 (susceptible to leaf scald) infected with *Xa*. In LCP85-384, 3 genes (*ShnsLTPI.8/I.10/Gb.1*) were significantly upregulated by 1.3–3.2-fold at all time points (particularly 72 hpi) relative to the control (0 hpi), whereas in ROC20 these same three genes were significantly downregulated. *ShnsLTPIV.3*, *MCOs* (Cluster-4871.429093), and *POD* (Cluster-43374.0) genes had variable expression patterns in LCP85-384, but were dramatically increased by 1.6–10.5-fold in ROC20 at all time points. Transcript levels of two genes (*ShnsLTPGa.4/Ge.3*) and the *KCS10* gene (Cluster-4871.251321) decreased compared to the control (0 hpi) in both cultivars over 24–72 hpi. The *ShnsLTPVIII.1* gene had fluctuating expression patterns in both cultivars after *Xa* infection (Figure 6).

### ShnsLTP expression patterns under salicylic acid treatment

**FIGURE 7:**
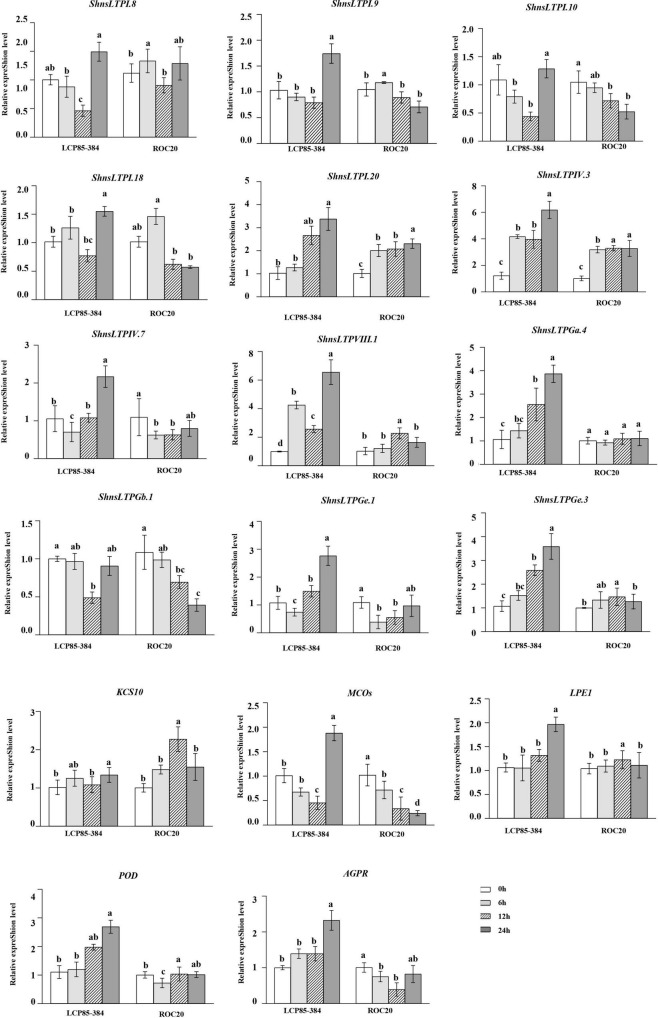
Transcript expression levels of 12 *ShnsLTPs* and 5 interacting protein genes in LCP85-384 (resistant to leaf scald) and ROC20 (susceptible to leaf scald) sugarcane cultivars after exogenous salicylic acid treatment. The bars indicate mean ± SD (*n* = 9). The same letters indicate no significant difference (*p* > 0.05).

Under SA treatment, all 17 *ShnsLTPs* except for *ShnsLTPGb.1* were significantly upregulated with an increase of 1.2–6.6-fold in LCP85-384 at 72 hpt. Six *ShnsLTPs* (*ShnsLTPI.8/I.9/I.10/I.18/IV.7/Ge.1*) and *MCOs* gene (Cluster-4871.429093) were downregulated in LCP85-384 soon after treatment (24 and/or 48 h). *ShnsLTPGb.1* expression was significantly depressed at 48 hpt, but was unchanged at 24 and 72 hpt. Notably, transcript levels for five *ShnsLTPs* (*ShnsLTPI.20/IV.3/VIII.1/Ga.4/Ge.3*) and the *AGPR* gene (Cluster-4871.162409) were continuously elevated with an increase ranging from 1.4- to 6.6-fold in LCP85-384 across all time points (Figure 7). On the other hand, five *ShnsLTP* genes (*ShnsLTPI.10/IV.7/Ga.4/Gb.1/Ge.1*) and two protein genes (*MCOs*, Cluster-4871.429093 and *AGPR*, Cluster-4871.162409) were significantly downregulated or showed no significant change, whereas two *ShnsLTP*s (*ShnsLTPI.20/IV.3*) and the *KCS10* gene (Cluster-4871.251321) were upregulated with an increase from 1.5- to 3.3-fold in ROC20 across all time points. *ShnsLTPIII.1/Ge.3* and *LPE1* expression was upregulated at 48 hpt, but had no significant change at either 24 or 72 hpt. Meanwhile, *ShnsLTPI.8/I.9/I.18* and *POD* gene exhibited fluctuating expression patterns in ROC20 at 24–72 hpt (Figure 7).

## Discussion

The nsLTPs are encoded by a complex of multigene families that have varying numbers of genes and carry out key biological functions in a wide of range of plants, such as *Arabidopsis*, *Oryza sativa*, and *Triticum aestivum* (Boutrot et al., 2008), *S. bicolor* and *Zea mays* (Wei and Zhong, 2014), *G. hirsutum* (Li et al., 2016), *Sesamum indicum* (Song et al., 2021), *Nicotiana sylvestris* and *Nicotiana tomentosiformis* (Yang et al., 2022), *Nicotiana tabacum* (Yang et al., 2022), and *B. napus* (Liang et al., 2022; Xue et al., 2022). The number of nsLTP family genes in plants varied from 13 *VvnsLTPs* in *Vitis vinifera* (Kobayashi et al., 2011) to 461 *TansLTPs* in *T. aestivum* (Kouidri et al., 2018; Fang et al., 2020). These genes were systematically identified at a genome-wide level. Highly variable numbers of nsLTPs were also present between plant genus members such as *N. sylvestris* (50) vs. *N. tabacum* (100) (Yang et al., 2022) and *Brassica rapa* (127) vs. *B. napus* (283). In this study, we identified 71 nsLTP genes with 66 alleles in *S. spontaneum* AP85-441; Type I had largest number of nsLTP genes (22). Recently, de Oliveira Silva et al. (2022) used 16 sequences of Type I LTPs from sorghum (*S. bicolor* L. Moench) (Wang et al., 2012) as bait to search for nsLTP members in *S. spontaneum* and identified 21 gene members of nsLTP Type I. High variability of nsLTP family members among different plant species might be due to different polyploidization or the duplication of entire genomes during plant evolution (Van de Peer et al., 2017). In addition, our data demonstrated that gene duplication, which is an important mechanism for acquiring new genes (Magadum et al., 2013; Liang et al., 2022), of these family genes was a common occurrence in the allopolyploid sugarcane.

Our results revealed that various *ShnsLTPs* played an opposing role in responses to *X. albilineans* infection. For example, *ShnsLTPI.8/I.10* might act as positive regulators, whereas *ShnsLTPGa.4/Ge.3* acted as negative regulators. Previous studies documented positive regulatory roles for different nsLTP family genes in different crops under pathogenic stress. The loss-of-function mutant *atltpI-4* (homolog of *ShnsLTPI.8/I.10*) displayed reduced crown gall formation associated with *Agrobacterium tumefaciens* pathogenesis (Deeken et al., 2016). Significantly enhanced levels of transcripts for the nsLTP 2-like gene (homolog of *ShnsLTPI.18*) were seen in tomato infected with *Cladosporium fulvum* (Jia et al., 2019). Additionally, *AtLTP4.4* (homolog of *ShnsLtpII.2*) positively modulated resistance of transgenic wheat to *F. graminearum* by inhibiting oxidative stress induced by deoxynivalenol (DON), a virulence factor produced by this fungus (McLaughlin et al., 2021). *StLTP10* (homolog of *ShnsLTPI.19*) enhanced resistance of potato to *P. infestans* by enhancing expression of reactive oxygen species (ROS) scavenging and defense-related genes (Wang et al., 2021). Overexpression of *NtLTPI.4/III.11* in tobacco enhanced tolerance to *Ralstonia solanacearum* (Yang et al., 2022). Notably, expression of *ShnsLTPI.8* (*Sspon.07G0018180-2B*) and *ShnsLTPI.10* (*Sspon.07G0018180-3C*) was upregulated in sugarcane infected with *A. avenae* (de Oliveira Silva et al., 2022).

On the other hand, some nsLTP family genes were previously shown to be involved in negative modulation of biotic stress. For instance, *NbLTP1* (homolog of *ShnsLTPI.19*) from *N. benthamiana* contributed to accumulation of bamboo mosaic virus that possibly resulted from the regulation of lipid-binding activity of NbLTP1 by calmodulin binding or phosphorylation (Chiu et al., 2020). In potato, *StLTP6* (homolog of *ShnsLTPI.20*) promoted viral infection by potato virus Y and S by inhibiting expression of multiple genes involved in the RNA silencing pathway (Shang et al., 2022). Interestingly, *GhnsLTPsA10* from cotton (*G. hirsutum*) negatively mediated insect (aphid and bollworm) resistance, yet positively enhanced resistance to fungi (*Verticillium dahliae* and *Fusarium oxysporum* f. sp. *vasinfectum*) in transgenic *Arabidopsis* and silenced cotton by modulating phenylpropanoid homeostasis and/or ROS accumulation (Chen et al., 2021). Recently, de Oliveira Silva et al. (2022) demonstrated that *SsnsLtpI.3* (*Sspon.03G0003670-4D*) and *SsnsLtpV.4* (*Sspon.05G0025940-2C*) play a negative regulatory role in response of sugarcane to *A. avenae* infection. Overall, functional redundancy and divergence within one gene family in plants, particularly sugarcane, which is a complex polyploidy crop, is frequent. Once polyploidy was established, the unique retention profile of duplicated genes might explain a general increase in biological complexity (Van de Peer et al., 2017).

Production of endogenous SA is induced after pathogen infection or exogenous SA application to plants. The corresponding SA signaling pathway is activated to induce expression of defense-related genes (Ding and Ding, 2020). Sufficient evidence indicated that nsLTPs are involved not only in resistance to biotic stresses but also actively respond to abiotic stresses such as SA treatment (Yang et al., 2022). Our findings showed that the majority of *ShnsLTPs* were upregulated in LCP85-384 at later stages (72 hpt) of SA stress, suggesting that they might play a positive role in SA-trigged responses. In particular, *ShnsLTPIV.3* expression was dramatically upregulated across all time points in both sugarcane cultivars with exogenous SA treatment. Conversely, some *ShnsLTPs* (e.g., *ShnsLTPI.10/Gb.1*) might play negative regulators in sugarcane trigged by SA stress, which is consistent with previous findings that *NtLTPI.1* (homolog of *ShnsLTPI.19*) (Jin et al., 2015) and *NtLTPI.34/NtLTPVIII.1* (Yang et al., 2022) in tobacco are strongly induced by SA. In contrast, *ScNsLTP* (homolog of *ShnsLtpII.1*) in sugarcane was inhibited by exogenous SA and downregulated across all time points, as part of the antagonistic regulation involving the MeJA signaling molecule (Chen et al., 2021). In *Arabidopsis*, *LTP3* (homolog of *ShnsLTPI.20*) expression is induced by pathogens and ABA and acts as a negative regulator in SA accumulation independently of ABA-independent manner (Gao et al., 2016). Additionally, the expression of key marker genes (*PR1/2/5*) associated with the SA signaling pathway was increased in *StLTP10* (homolog of *ShnsLTPI.19*)-overexpressing plants compared with wild type and *StLTP10*-RNAi plants, indicating that this gene may participate in SA-mediated defense pathways (Wang et al., 2021). However, little is known about endogenous SA levels and signaling or SA-mediated defense responses in sugarcane trigged by *Xa* infection.

## Conclusion

We systematically identified 71 *SsnsLTP* genes (66 alleles) in the AP85-441 *S. spontaneum* genome and demonstrated that these genes have diverse ECM structural features and can be classified into 13 nsLTP classes. Based on transcript expression patterns of 12 *ShnsLTPs* in *Xa*-infected plants as determined by RT-qPCR, we proposed that the *ShnsLTPI.8/10/Gb.1* genes might play a positive regulatory role, whereas *ShnsLTPGa.4/Ge.3* genes would act as a negative regulator during *Xa*-infection. In addition, following SA treatment *ShnsLTPIV.3/VIII.1* genes might be positive regulators, whereas *ShnsLTPGb.1* would be a negative regulator. However, the mechanisms by which these genes function in host defense responses to *Xa* infection, particularly those involving the ROS production-scavenging system, and during activation of the SA pathway require further exploration. To better understand the function of *ShnsLTPs*, genetic engineering can be applied to develop transgenic sugarcane that overexpress genes associated with tolerance or knockout of suspectable genes by gene editing techniques such as CRISPR-Cas9.

## Data availability statement

The datasets presented in this study can be found in online repositories. The names of the repository/repositories and accession number(s) can be found in the article/Supplementary material.

## Author contributions

JL and S-JG: conceptualization and writing—review and editing. JL and YS: writing—original draft preparation. JL and J-YM: bioinformatics analysis. J-YZ, H-YF, and M-TH: experiments and data reduction. S-JG: supervision, funding acquisition, and project administration. All authors have read and agreed to the final version of the manuscript.

## Footnotes

1
https://www.life.illinois.edu/ming/downloads/Spontaneum_genome/

2
http://pfam.xfam.org/

3
https://blast.ncbi.nlm.nih.gov/Blast.cgi

4
https://mapchart.net/

5
http://web.expasy.org/compute_pi/

6
https://www.bioinformatics.org/sms/prot_mw.html

7
http://web.expasy.org/protparam/

8
http://gsds.gao-lab.org/

9
https://meme-suite.org/meme/

10
http://www.string-db.org

11
http://www.premierbiosoft.com/primerdesign/
