# Supplementary material for: Systematic and functional analysis of non-specific lipid transfer protein family genes in sugarcane under Xanthomonas albilineans infection and salicylic acid treatment
Source: Front Plant Sci. 2022 Oct 5;13:1014266. doi: 10.3389/fpls.2022.1014266 (PMC9581186; doi:10.3389/fpls.2022.1014266)
Supplement: Supplementary file 1 [file Data_Sheet_1.docx]

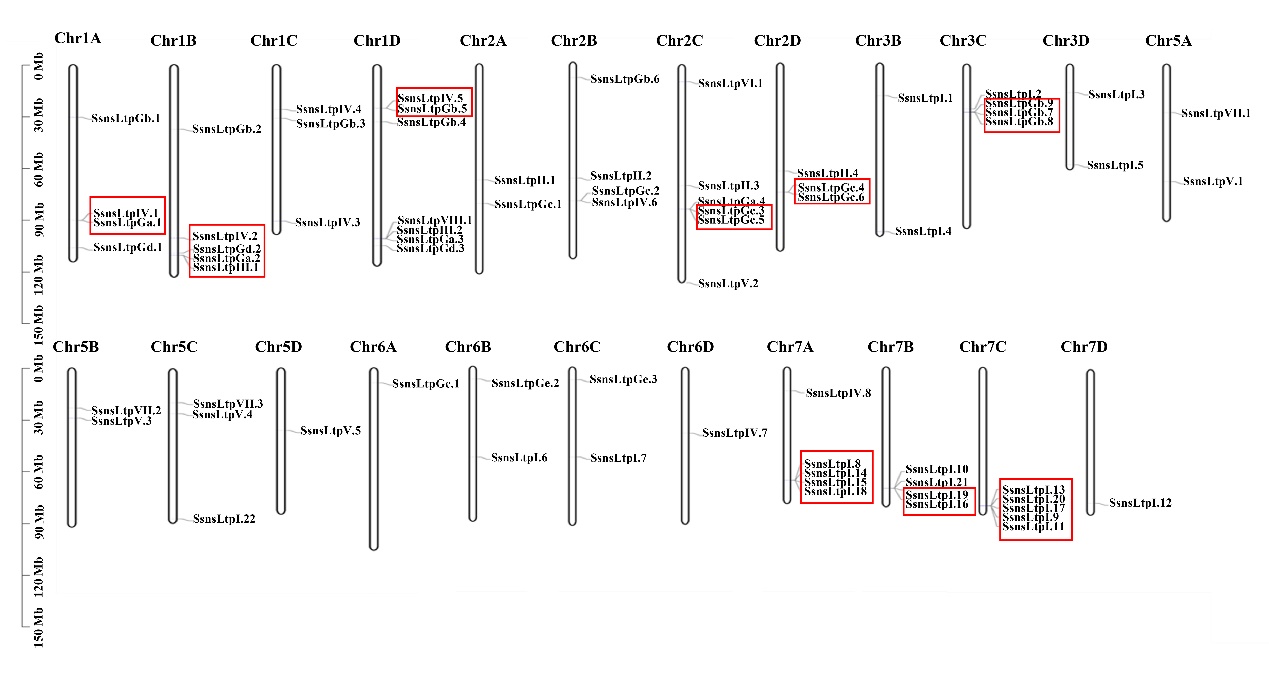


**Supplemental Figure 1.** Chromosome location of the 71 *SsnsLTP* genes in *S. spontaneum* genome of AP85-441*.* The genes with tandem gene replication are shown in a red box.

**Supplemental Table 1.** Primer pairs used for RT-qPCR analysis in this study.

| **No** | **Target gene** | **Forward primer (5'-3')** | **Reverse primer (5'-3')** | **Tm (^o^C)** | **Amplicon size (bp)** |
| --- | --- | --- | --- | --- | --- |
| 1 | *GAPDH* | CACGGCCACTGGAAGCA | TCCTCAGGGTTCCTGATGCC | 58 | 110 |
| 2 | *SsnsLTPI.8* | GTGCGCAGCCTCAACAGC | GCGACGGTGCCCATGTTGAC | 59 | 110 |
| 3 | *SsnsLTPI.9* | GTGCGCAGCCTCAACAGC | GCGACGGTGCCCATGTTGAC | 60 | 110 |
| 4 | *SsnsLTPI.10* | CGTCTCCGTGGGCTTCCC | GCTTCATCACGTGCGCTGGTA | 58 | 102 |
| 5 | *SsnsLTPI.18* | CGCCTGCAACTGCCTCAAGA | GTCGGTGGAGGTGCTGATGG | 58 | 121 |
| 6 | *SsnsLTPI.20* | GGCGTCAGGAGCCTCAACTC | GCTGACGCCGCACTTGGAG | 61 | 141 |
| 7 | *SsnsLTPIV.3* | TTGCGTCGTGTCAGCTTTCG | GCGACCGTAGGAATCACCGA | 59 | 103 |
| 8 | *SsnsLTPIV.7* | AGGCTCACCATCTCCGTCAA | GAGTTCGAGCTCCCGCAATG | 58 | 158 |
| 9 | *SsnsLTPVIII.1* | CCATCATGGCGTCCATGTCC | TAGCTGGCTAGCTCCGATCC | 58 | 123 |
| 10 | *SsnsLTPGa.4* | GTCGACTCGTTCCCGGAGATG | CCACACGACGCGACAGAGAA | 59 | 175 |
| 11 | *SsnsLTPGb.1* | ACCGAAGCACCTGCACCATC | CGGTCGCCGTCATTGGAGAA | 60 | 103 |
| 12 | *SsnsLTPGe.1* | CGACGTCAGCAACTGCAAGG | CGTGGGCGTCGTCTTGGAC | 60 | 109 |
| 13 | *SsnsLTPGe.3* | ATTGAAGCCGGAGCGGTGAG | ACGTGGTGTGCGCAACTGTA | 60 | 90 |
| 14 | *Nucleobase-ascorbate transporter lpe1* | GGTGGGACAAGTTCCGGTCA | TGCCACCACCAGAGAACAGG | 59 | 135 |
| 15 | *N-acetyl-γ-glutamyl phosphate reductase* | CCTCACTTGAGAACGCTGGA | GTAGTCGGAAGTCCGCTGAG | 57 | 151 |
| 16 | *The multicopper oxidase family* | GGCCGACGGTGAAGAAGAGG | TCGAGTACGACTGCGGTTGC | 60 | 157 |
| 17 | *Peroxidase* | TCAGCAAGCTCAAGGAGGTG | TCTCGAAGTAGGCGTTGTCG | 57 | 82 |
| 18 | *3-ketoacyl-CoA Enzyme 10* | TGCTGGAGGTGTTGCCGAAG | AGCACCTGCAGAGCAACCTG | 60 | 77 |

**Supplementary Table 2** The Physio-chemical characteristics of nsLTP family genes in *Saccharum spontaneum* AP85-441.

| **Name** | **Gene ID** | **Number of amino acids (aa)** | **Molecular weight (Da)** | **Isoelectric point** | **Instability index** | **Aliphatic index** | **Hydrophilicity** |
| --- | --- | --- | --- | --- | --- | --- | --- |
| SsnsLTPI.1 | Sspon.03G0003670-2B | 121 | 12256.43 | 9.06 | 45.41 | 89.67 | 0.4 |
| SsnsLTPI.2 | Sspon.03G0003670-3C | 289 | 30759.47 | 8.71 | 51.41 | 76.68 | 0.09 |
| SsnsLTPI.3 | Sspon.03G0003670-4D | 123 | 12469.71 | 9.22 | 44.15 | 91.38 | 0.42 |
| SsnsLTPI.4 | Sspon.03G0037230-1B | 121 | 12068.16 | 8.5 | 44.12 | 93.06 | 0.69 |
| SsnsLTPI.5 | Sspon.03G0037230-2D | 121 | 12038.07 | 8.5 | 43.65 | 93.06 | 0.67 |
| SsnsLTPI.6 | Sspon.06G0024230-1B | 122 | 12741.95 | 8.67 | 43.7 | 85.82 | 0.16 |
| SsnsLTPI.7 | Sspon.06G0024230-2C | 126 | 13123.29 | 8.12 | 45.92 | 83.81 | 0.19 |
| SsnsLTPI.8 | Sspon.07G0018120-1A | 131 | 13292.39 | 9.2 | 45.36 | 90.15 | 0.38 |
| SsnsLTPI.9 | Sspon.07G0018120-1P | 145 | 14610.73 | 9.16 | 34.88 | 83.52 | 0.21 |
| SsnsLTPI.10 | Sspon.07G0018120-2B | 145 | 14610.73 | 9.16 | 34.88 | 83.52 | 0.21 |
| SsnsLTPI.11 | Sspon.07G0018120-3C | 124 | 12200.15 | 9.58 | 39.32 | 90.56 | 0.51 |
| SsnsLTPI.12 | Sspon.07G0018120-4D | 146 | 14668.82 | 9.16 | 34.8 | 86.3 | 0.26 |
| SsnsLTPI.13 | Sspon.07G0018130-2C | 119 | 11584.24 | 9.1 | 46.86 | 87.98 | 0.43 |
| SsnsLTPI.14 | Sspon.07G0018160-1A | 132 | 13027.16 | 8.81 | 33.95 | 88.18 | 0.39 |
| SsnsLTPI.15 | Sspon.07G0018170-1A | 122 | 11651.48 | 9.1 | 43.83 | 85.74 | 0.53 |
| SsnsLTPI.16 | Sspon.07G0018170-2B | 221 | 23059.45 | 9.52 | 52.61 | 74.71 | -0.05 |
| SsnsLTPI.17 | Sspon.07G0018170-3C | 122 | 11664.48 | 9.1 | 43.21 | 85.74 | 0.51 |
| SsnsLTPI.18 | Sspon.07G0018180-1A | 119 | 11602.25 | 9.12 | 36.05 | 87.9 | 0.48 |
| SsnsLTPI.19 | Sspon.07G0018180-2B | 118 | 11583.25 | 9.3 | 32.79 | 86.19 | 0.41 |
| SsnsLTPI.20 | Sspon.07G0018180-3C | 119 | 11602.25 | 9.12 | 36.05 | 87.9 | 0.48 |
| SsnsLTPI.21 | Sspon.07G0028840-1B | 121 | 11753.44 | 9.1 | 34.38 | 92.98 | 0.5 |
| SsnsLTPI.22 | Sspon.07G0028840-2C | 122 | 11972.86 | 9.27 | 49.48 | 96.97 | 0.58 |
| SsnsLTPII.1 | Sspon.02G0020560-1A | 114 | 11880.84 | 6.03 | 48.62 | 85 | 0.21 |
| SsnsLTPII.2 | Sspon.02G0020560-2B | 105 | 10895.75 | 6.01 | 53.78 | 82.95 | 0.35 |
| SsnsLTPII.3 | Sspon.02G0050950-1C | 116 | 11893.73 | 6.08 | 74.62 | 75.95 | 0.16 |
| SsnsLTPII.4 | Sspon.02G0050950-2D | 111 | 11371.08 | 5.09 | 78.09 | 73.24 | 0.13 |
| SsnsLTPIII.1 | Sspon.01G0048690-1B | 109 | 10999.89 | 7.49 | 43.71 | 102.11 | 0.64 |
| SsnsLTPIII.2 | Sspon.01G0048690-2D | 109 | 10999.89 | 7.49 | 43.71 | 102.11 | 0.64 |
| SsnsLTPIV.1 | Sspon.01G0025430-1A | 208 | 22069.28 | 10.08 | 56.84 | 82.26 | 7.45 |
| SsnsLTPIV.2 | Sspon.01G0025440-2B | 224 | 21945.8 | 8.15 | 61.61 | 78.21 | 0.23 |
| SsnsLTPIV.3 | Sspon.01G0025440-3C | 216 | 21772.56 | 7.84 | 63.14 | 75.14 | 0.13 |
| SsnsLTPIV.4 | Sspon.01G0052710-1C | 146 | 14335.91 | 7.49 | 48.59 | 107.19 | 0.64 |
| SsnsLTPIV.5 | Sspon.01G0052710-1T | 194 | 19062.35 | 8.66 | 49.12 | 103.92 | 0.55 |
| SsnsLTPIV.6 | Sspon.02G0041940-1B | 162 | 15419.45 | 8.71 | 42.2 | 79.07 | 0.28 |
| SsnsLTPIV.7 | Sspon.06G0034090-1D | 162 | 15354.38 | 8.5 | 83.71 | 81.48 | 0.37 |
| SsnsLTPIV.8 | Sspon.07G0005420-1A | 142 | 14085.38 | 4.41 | 60.76 | 92.89 | 0.52 |
| SsnsLTPV.1 | Sspon.05G0016500-1A | 110 | 11246.15 | 8.49 | 42.25 | 89.73 | 0.39 |
| SsnsLTPV.2 | Sspon.05G0016500-2C | 110 | 11270.22 | 8.49 | 38.09 | 94.09 | 0.4 |
| SsnsLTPV.3 | Sspon.05G0025940-1B | 261 | 27399.89 | 9.32 | 55.38 | 85.21 | 0.08 |
| SsnsLTPV.4 | Sspon.05G0025940-2C | 130 | 13015.1 | 8.36 | 56.12 | 80.54 | 0.27 |
| SsnsLTPV.5 | Sspon.05G0025940-3D | 338 | 34742.52 | 9.37 | 58.09 | 78.67 | 0.04 |
| SsnsLTPVI.1 | Sspon.02G0033810-2C | 186 | 18949.89 | 9.53 | 54.6 | 80.43 | 0.14 |
| SsnsLTPVII.1 | Sspon.05G0009800-1A | 221 | 22651.29 | 8.67 | 60.08 | 78.69 | 0.16 |
| SsnsLTPVII.2 | Sspon.05G0009800-2B | 192 | 19681.39 | 4.74 | 48.62 | 82.97 | 0.19 |
| SsnsLTPVII.3 | Sspon.05G0009800-3C | 190 | 19300.6 | 8.45 | 52.68 | 87.89 | 0.44 |
| SsnsLTPVIII.1 | Sspon.01G0048670-2D | 714 | 76418.3 | 6.04 | 49.78 | 80.14 | -0.02 |
| SsnsLTPGa.1 | Sspon.01G0025440-1A | 158 | 14916.79 | 7.49 | 59.18 | 81.84 | 0.37 |
| SsnsLTPGa.2 | Sspon.01G0048680-1B | 187 | 18023.67 | 6.23 | 56.75 | 79.63 | 0.38 |
| SsnsLTPGa.3 | Sspon.01G0048680-2D | 187 | 18051.73 | 6.23 | 56.75 | 80.64 | 0.39 |
| SsnsLTPGa.4 | Sspon.02G0041940-2C | 179 | 16932.31 | 8.71 | 39.35 | 86.26 | 0.49 |
| SsnsLTPGb.1 | Sspon.01G0011020-1A | 178 | 16841.44 | 8.36 | 59.27 | 90.34 | 0.67 |
| SsnsLTPGb.2 | Sspon.01G0011020-2B | 176 | 16713.31 | 8.36 | 58.79 | 90.8 | 0.68 |
| SsnsLTPGb.3 | Sspon.01G0011020-3C | 178 | 16875.46 | 8.36 | 59.97 | 88.15 | 0.67 |
| SsnsLTPGb.4 | Sspon.01G0011020-4D | 176 | 16681.25 | 8.36 | 59.39 | 92.44 | 0.68 |
| SsnsLTPGb.5 | Sspon.01G0052710-2D | 194 | 19062.35 | 8.66 | 49.12 | 103.92 | 0.55 |
| SsnsLTPGb.6 | Sspon.02G0033810-1B | 210 | 20877.5 | 9.06 | 52.74 | 101 | 0.64 |
| SsnsLTPGb.7 | Sspon.07G0005420-1P | 189 | 18445.41 | 5.83 | 51.23 | 91.59 | 0.52 |
| SsnsLTPGb.8 | Sspon.07G0005420-2C | 189 | 18445.41 | 5.83 | 51.23 | 91.59 | 0.52 |
| SsnsLTPGb.9 | Sspon.07G0005420-2P | 189 | 18445.41 | 5.83 | 51.23 | 91.59 | 0.52 |
| SsnsLTPGc.1 | Sspon.02G0023650-1A | 199 | 19624.73 | 8.52 | 64.01 | 92.86 | 0.45 |
| SsnsLTPGc.2 | Sspon.02G0023650-1T | 202 | 19894.03 | 8.52 | 64.16 | 91.98 | 0.44 |
| SsnsLTPGc.3 | Sspon.02G0023650-3C | 202 | 19825.91 | 8.52 | 62.78 | 91.04 | 0.44 |
| SsnsLTPGc.4 | Sspon.02G0023650-4D | 202 | 19935.13 | 8.77 | 61.82 | 93.42 | 0.46 |
| SsnsLTPGc.5 | Sspon.02G0023670-3C | 209 | 20371.69 | 7.52 | 63.57 | 95.02 | 0.52 |
| SsnsLTPGc.6 | Sspon.02G0023670-4D | 214 | 20981.33 | 6.08 | 66.37 | 85.05 | 0.46 |
| SsnsLTPGd.1 | Sspon.01G0030470-1A | 178 | 18380.52 | 8.37 | 31.73 | 83.09 | 0.35 |
| SsnsLTPGd.2 | Sspon.01G0030470-2B | 186 | 19079.25 | 8.06 | 29.02 | 81.61 | 0.33 |
| SsnsLTPGd.3 | Sspon.01G0030470-3D | 208 | 21185.6 | 8.21 | 36.67 | 76.3 | 0.32 |
| SsnsLTPGe.1 | Sspon.06G0002800-1A | 181 | 18805.47 | 8.05 | 54.95 | 87.51 | 0.15 |
| SsnsLTPGe.2 | Sspon.06G0002800-2B | 161 | 16703.1 | 8.06 | 66.09 | 88.07 | 0.21 |
| SsnsLTPGe.3 | Sspon.06G0002800-3C | 181 | 18918.64 | 8.36 | 59.18 | 88.07 | 0.12 |
